# Supplementary figures and images for: NF-κB c-Rel Is Dispensable for the Development but Is Required for the Cytotoxic Function of NK Cells
Source: Front Immunol. 2021 Apr 29;12:652786. doi: 10.3389/fimmu.2021.652786 (PMC8116710; doi:10.3389/fimmu.2021.652786)

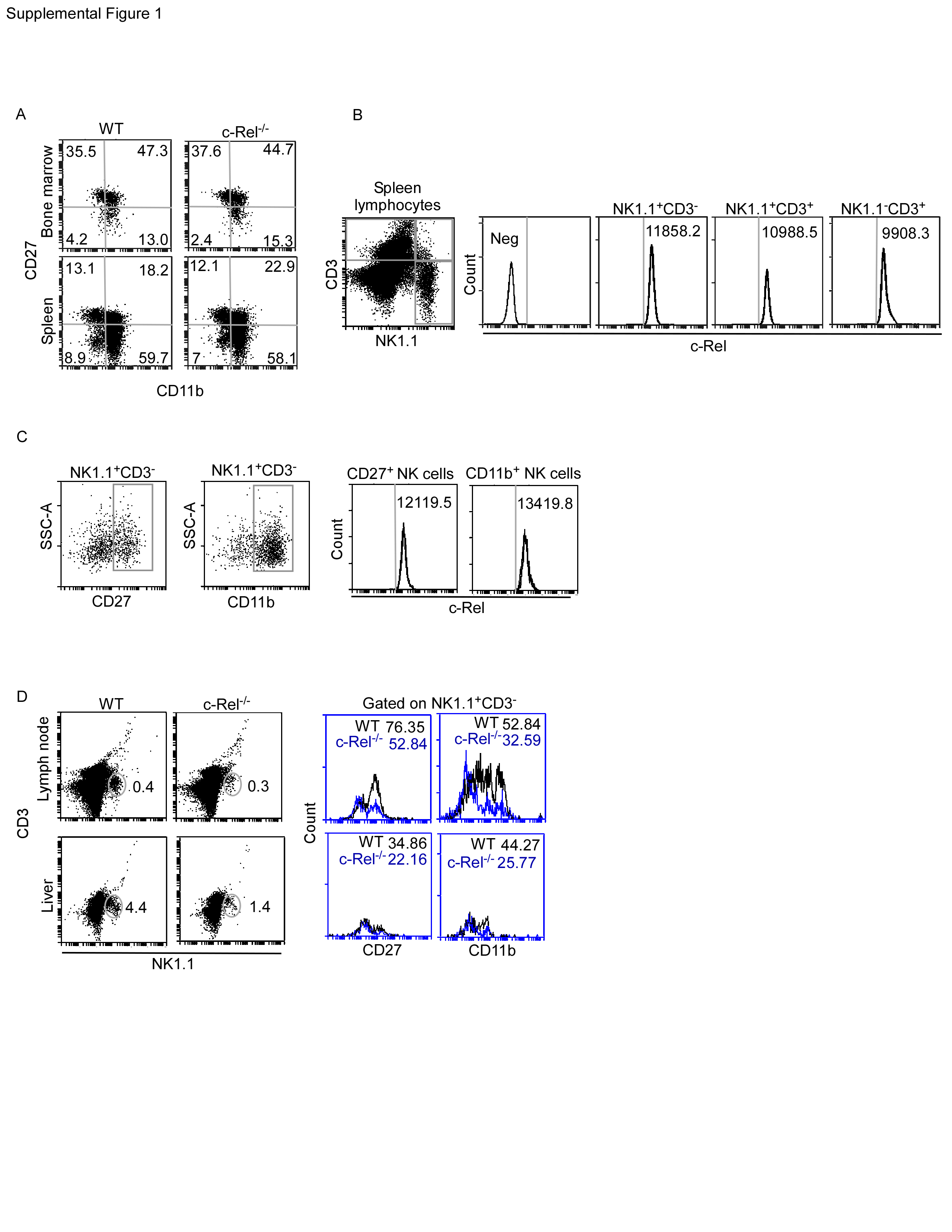

Supplement: Supplementary Figure 1 — Related to Figure 1 : c-Rel expression in NK cells. Single suspension cells from bone marrow and spleen of WT and c-Rel-/- mice were analyzed via flow cytometry. (A) Dot plots of CD11b and CD27 expression on gated CD3-NK1.1+ NK cells from WT and c-Rel-/- mice (n=8-13). Total splenocytes from WT mice were stained intracellularly for c-Rel and analyzed via flow cytometry. (B) Histograms of c-Rel expression on gated CD3-NK1.1+ (NK), CD3+NK1.1+ (NKT), and CD3+NK1.1- (T) splenic cells (n=4). (C) Histograms of c-Rel expression on gated CD11b+ or CD27+ NK cells. Experiment was repeated four times with 4 mice of each genotype (n=4). Isotype IgG served as negative control for staining. (D) Dot plots representative of percentages of gated CD3-NK1.1+ NK cells (left) or histograms (right) showing the percentages of CD27+ and CD11b+ NK cells in WT (black) and c-Rel-/- (Blue) mice in the indicated organs. [file Image_1.tif]

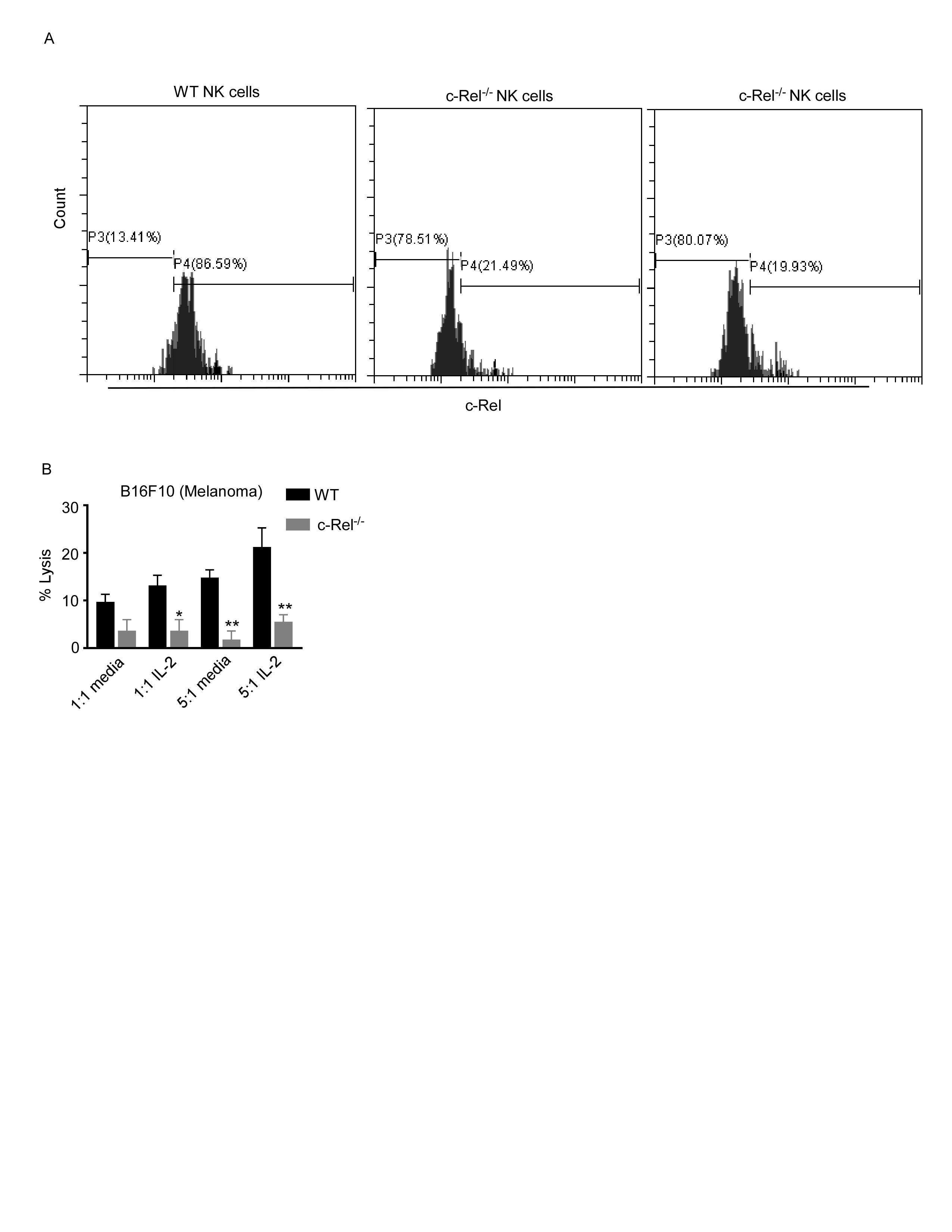

Supplement: Supplementary Figure 2 — Related to Figure 2 : Flow cytometry analysis of c-Rel expression in WT and c-Rel-/- mice. Total splenocytes from WT and c-Rel-/- mice were stained intracellularly for c-Rel and analyzed via flow cytometry. (A) c-Rel expression in WT and c-Rel-/- mice (n=2). (B) Percent lysis of melanoma (B16F10) murine tumor cells co-cultured with either media or IL-2 pretreated (24 hours) WT or c-Rel-/- NK cells for four hours at the indicated effector (E) target (T) ratio. Tumor cells alone was used as negative control and tumor cells lysed with detergent served as positive control. Data represents mean ± SEM of two independent experiments (n=4). Data were analyzed using Student’s t-test. *p<0.05; **p<0.01. [file Image_2.tif]

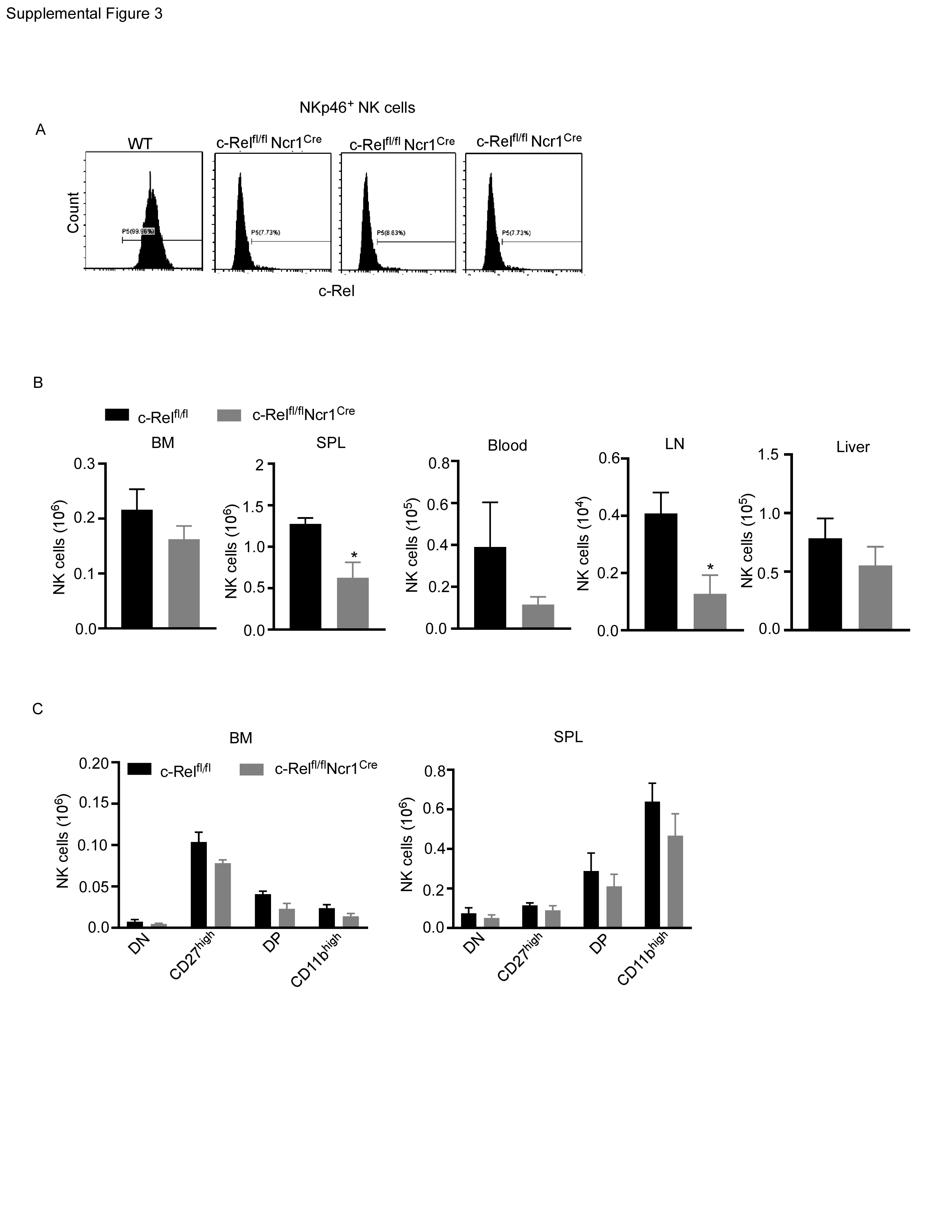

Supplement: Supplementary Figure 3 — Related to Figure 3 : Cell-intrinsic effect of c-Rel deficiency in NK cell numbers. NK cell specific c-Rel deficient (c-Relfl/flNcr1Cre) and c-Rel flox (c-Relfl/fl) mice were used to evaluate the cell-intrinsic effects of c-Rel deficiency in NK cells. (A) Flow cytometry expression analysis of c-Rel in NK cells from WT and c-Relfl/flNcr1Cre mice (n=3). (B) Total numbers of NK cells in the indicated organ of c-Relfl/fl mice and c-Relfl/flNcr1Cre. CD3-NKp46+ NK cells were analyzed for the expression of CD27 and CD11b maturation markers. (C) Total numbers of NK cell subsets from c-Relfl/fl mice and c-Relfl/flNcr1Cre mice. Each column represents the mean ± SEM of 3-4 mice. For details on calculations of NK cell total numbers please see Figure 1 legend. Data were analyzed by unpaired Student’s t-test. *p<0.05. [file Image_3.tif]

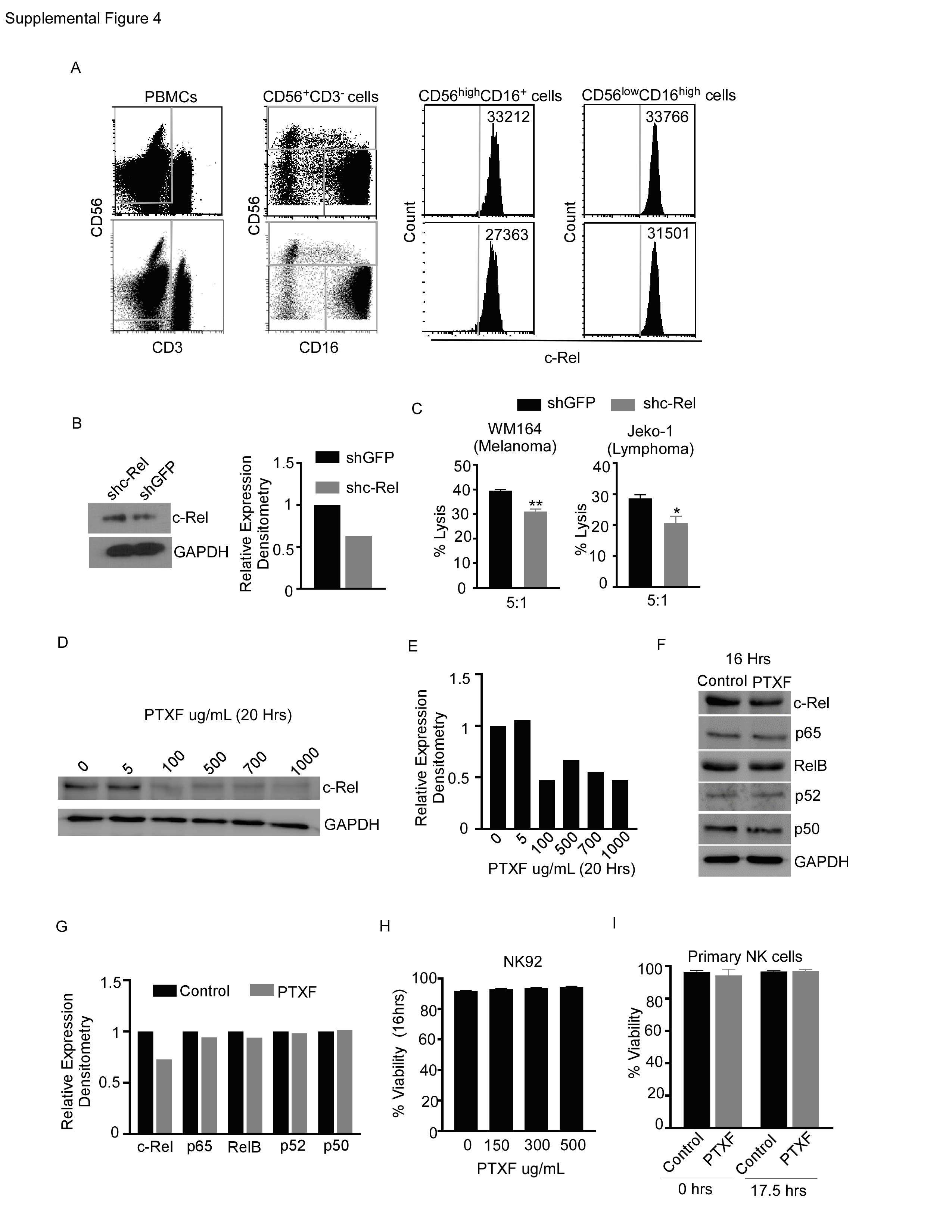

Supplement: Supplementary Figure 4 — Related to Figure 4 : Effect of PTXF on NK cell viability and NF-κB subunits. c-Rel expression in primary human NK cells was evaluated by intracellular staining for c-Rel followed by flow cytometry analysis. (A) Dot plots of NK cell gating strategy (left) and histograms of c-Rel MFI in the indicated NK cell subset. Data from two individual human donors. The human NK cell line (NK92) was transduced with shRNA lentiviral vectors containing either GFP (shGFP), a negative control or shRNA targeting c-Rel (shc-Rel) and analyzed for western blot. (B) Western blot analysis (left) and densitometry (right) of c-Rel expression 48 hours post puromycin selection. GAPDH was used as loading control. (C) Percentage of Propidium iodide (PI) positive tumor cells after 4-hour co-culture with either shGFP or shc-Rel transduced NK92 cells, measured via flow cytometry. Data represents mean ± SEM of three technical replicates. (D) Dose response of PTXF on c-Rel expression in primary NK cells treated with water (vehicle control) or Pentoxiphyline (PTXF) inhibitor for 20 hours. (E) Densitometry analysis of c-Rel expression in primary NK cells treated with water (vehicle control) or Pentoxiphyline (PTXF) inhibitor with the indicated concentrations for 20 hours. GAPDH was used as loading control. (F) Western blot analysis and (G) Densitometry of NF-κB subunits expression in primary NK cells treated with water (vehicle control) or Pentoxiphyline (PTXF) inhibitor (500 ug/mL) for 16 hours. (H, I) Percent viable NK92 and primary NK cells after treatment with either water (vehicle control) or Pentoxiphyline (PTXF) inhibitor (500 ug/mL) for the indicated time. Experiment represents three technical replicates. Data were analyzed using Student’s t-test. *p<0.05; **p<0.01; ****p<0.0001. [file Image_4.tif]

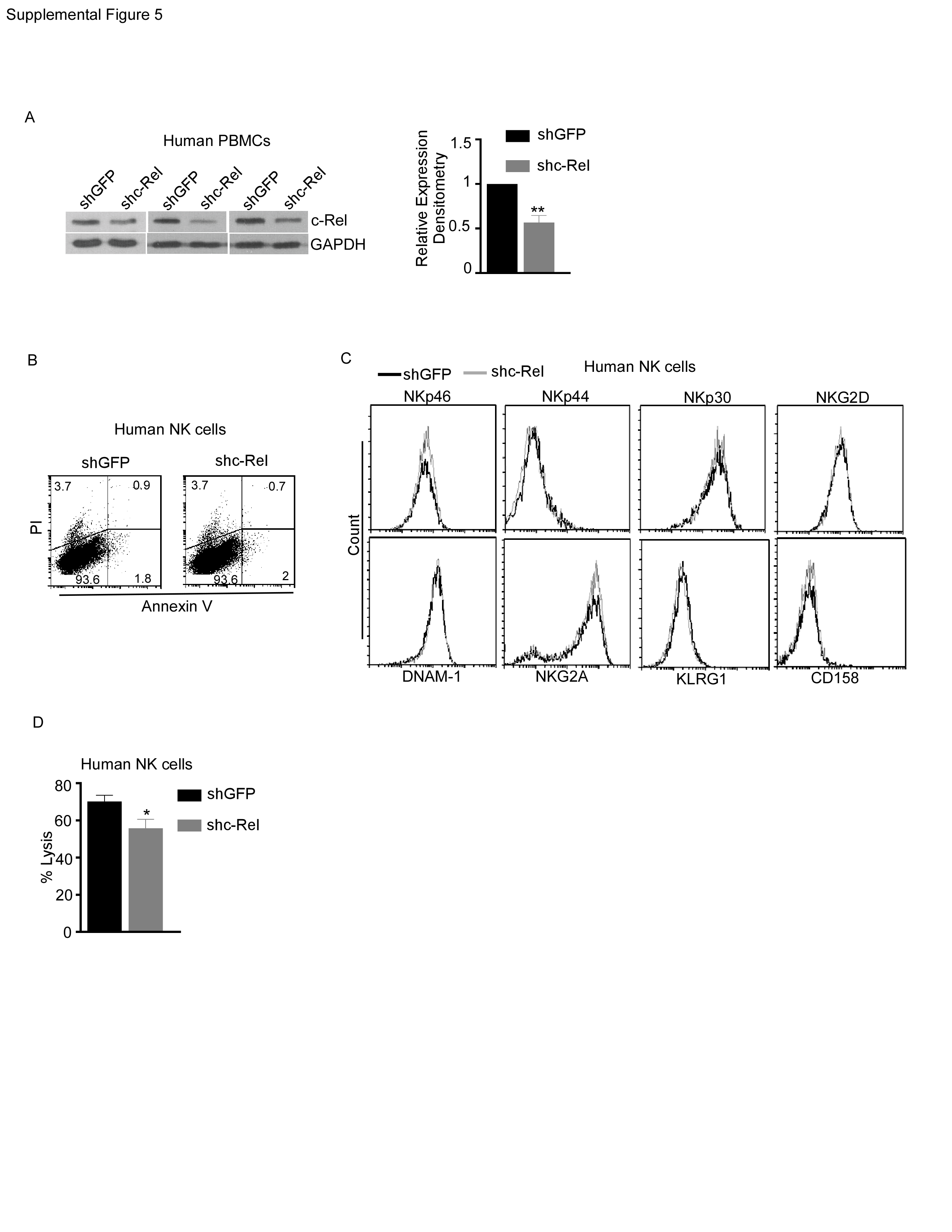

Supplement: Supplementary Figure 5 — Related to Figure 4 : c-Rel knockdown in human NK cells. To evaluate the expression of receptors important for NK cell anti-tumor activity, human PBMCs were transuded with shRNA lentiviral vectors containing either GFP (shGFP), a negative control or shRNA targeting c-Rel (shc-Rel) and analyzed for western blot. (A) Western blot (left) and densitometry (right) analysis of c-Rel in primary NK cells transduced with shGFP or shc-Rel (72 hours). Experiment represents 3 independent analysis. (B) Viability of primary NK cells post-transduction as determined by Annexin V and Propidium iodide staining. (C) Histogram of flow cytometry analysis of activating and inhibitory receptors in shGFP or sh-cRel transduced primary NK cells. (D) Percent lysis of melanoma WM164 human tumor cells co-cultured with either shGFP or shc-Rel primary NK cells for four hours at an effector (E) target (T) ratio of 2.5:1 as measured by calcein-AM release assay. Tumor cells alone was used as negative control and tumor cells lysed with detergent served as positive control. Data represents technical triplicates. Data were analyzed using unpaired Student’s t-test. *p<0.05. [file Image_5.tif]

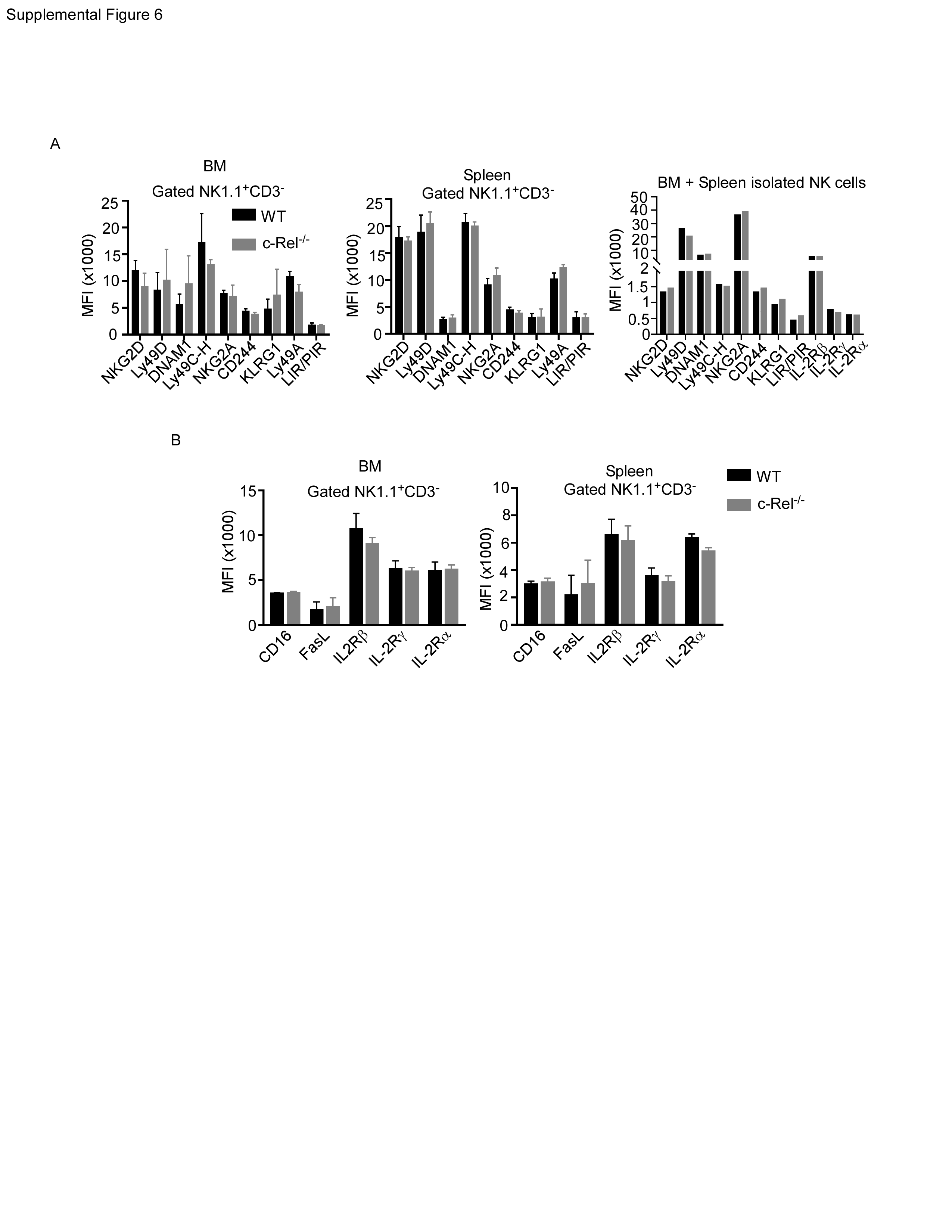

Supplement: Supplementary Figure 6 — Related to Figure 5 : Activating and inhibitory receptor expression in WT and c-Rel-/- NK cells. To evaluate the expression of receptors important for NK cell anti-tumor activity, single suspension cells were isolated from the bone marrow and spleen of c-Rel-/- mice and analyzed by flow cytometry. (A, B) Representative Mean Fluorescence Intensity (MFI) of gated NK cells expressing the indicated activating or inhibitory receptors from the bone marrow, the spleen or combine bone marrow and spleen. Data represents mean ± SEM of 2-3 independent experiments (n=2-3 per genotype) and were analyzed using unpaired Student’s t-test and all of these analysis in (A, B) were not significant with p>0.05. [file Image_6.tif]

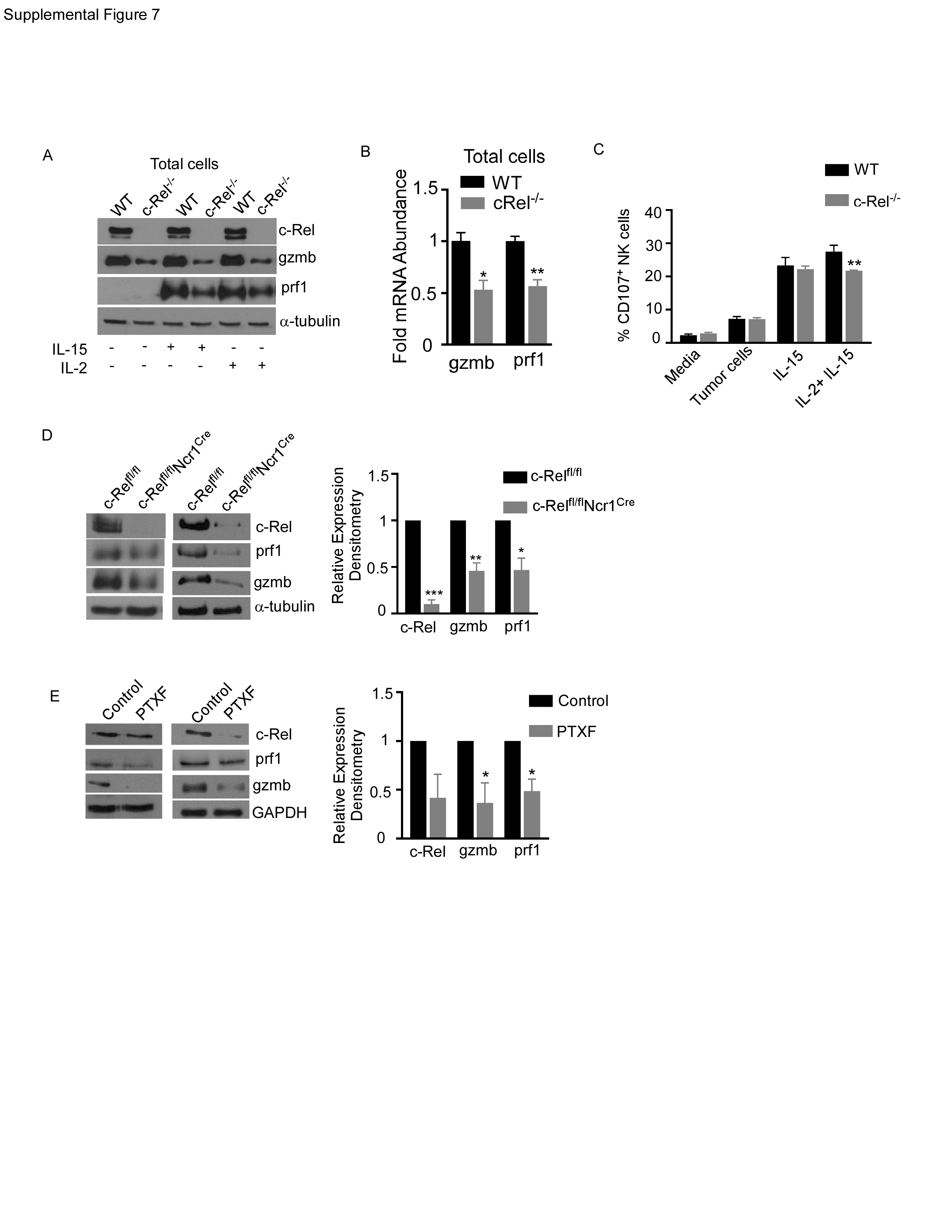

Supplement: Supplementary Figure 7 — Related to Figure 6 : Effect of c-Rel deficiency in perforin and granzyme b expression. The expression of cytotoxic mediators granzyme B (gzmb) and perforin (prf1) was evaluated in NK and total cells pooled from the bone marrow and spleen of WT and c-Rel-/- mice. (A) Western blot analysis of total lysates from total cells pooled from the spleen and bone marrow of WT or c-Rel-/- mice treated for 48 hours with the indicated cytokines (n=3-4). (B) PCR analysis of gzmb and prf1 expression in isolated total cells from the spleen and bone marrow of WT or c-Rel-/- mice (n=3 per group). (C) Percentage CD107+ WT or c-Rel-/- NK cells cultured with media alone (no tumor cells), tumor cells 8093 (4 hours) at a 1:1 effector: target ratio, IL-15 (tumor+IL-15), or the combination of IL-15 with IL-2 (tumor+IL-2+IL-15) for 24 hours. (D) Western blot (left) and densitometry (right) analysis of c-Rel, perforin, and granzyme b expression in cells (spleen and bone marrow) from c-Relfl/flNcr1Cre and c-Relfl/fl mice. Densitometry data represents mean of three independent experiments. (E) Western blot (left) and densitometry (right) analysis of c-Rel, perforin, and granzyme b expression in primary human NK cells treated with water or PTXF for 16 hours. Densitometry data represents mean of three independent experiments. Data were analyzed using Student’s t-test. *p<0.05; **p<0.01; ***p<0.001. [file Image_7.tif]
